# 

**Published:** 1895-12

**Authors:** 


### CONTENTS.

ORIGINAL ARTICLES:
- Tuberculosis. By W. B. NILES, D.V.M. . . . 781
- Control of Tuberculosis in Massachusetts. By FREDERICK H. OSGOOD, M.R.C.V.S. 791
- Work of Foreign Veterinarians in Tuberculosis. By DR. LEONARD PEARSON . 797
- Tuberculosis as Viewed by a Veterinarian. By H. D. GILL, V.S . . . 804
- The Extinction of Tuberculosis. By PROF. JAMES LAW . . . 808
- Castration of Cryptorchids. By G. W. BROWNING, V.S. . . . 817
- Arytenectomy for Roaring in the Horse. By DR. S. J. J. HARGER . . . 823

Extracts from Foreign Journals . . . 827

REPORTS OF CASES:
- Enlargement of the Near Hock in Animals Shown to the Roller for any Length of Time. By A. W. CLEMENT, V. S. . . . 829

LEGISLATION:
- New York State Veterinary License Law . . . 830
- Pennsylvania State Board of Veterinary Medical Examiners . . . 831

CONTROL WORK . . . 833

CORRESPONDENCE . . . 836

EDITORIALS:
- Prospectus for 1896 . . . 840
- Association Duty of the Hour . . . 841
- One More Word . . . 843
- Our December Number . . . 844
- Journal Change . . . 844
- Is This a Blunder? . . . 845

OBITUARIES—R. A. McLean—John Duane, Jr. . . . 847

REVIEW . . . 848

SOCIETY MEETINGS AND PROCEEDINGS:
- Missouri State Veterinary Medical Association—Massachusetts Veterinary Association—Missouri Valley Veterinary Medical Association—Montreal Veterinary Medical Association—Keystone Veterinary Medical Association . . 849–852

AMONG THE COLLEGES . . . 853

FOUND AMONG MANY PLACES AND SOURCES . . . 854

PERSONAL . . . 855

NEW INVENTIONS . . . 856

TITLE AND INDEX.

